# The First Ancient DNA Evidence of Zebu Husbandry in Thailand During the Prehistoric Through the Historic Periods

**DOI:** 10.3390/ani16121904

**Published:** 2026-06-19

**Authors:** Pornchanok Yensookjai, Suteera Prachumsarn, Noppasin Sangtubsorn, Yada Katanyuphan, Pee Boonleang, Pipad Krajaejun, Athiwat Wattanapituksakul, Wunrada Surat

**Affiliations:** 1 Department of Genetics, Faculty of Science, Kasetsart University, Bangkok 10900, Thailand; pornchanok.ye@ku.th (P.Y.); suteera.pra@ku.th (S.P.); noppasin.s@ku.th (N.S.); 2 Department of History, Faculty of Liberal Arts, Thammasat University, Pathum Thani 12120, Thailand; pipad_k@yahoo.com; 3 The Timing and the Cause of the Transition in Domestic Cattle Species in Thailand Project, Department of Genetics, Faculty of Science, Kasetsart University, Bangkok 10900, Thailand; essathiwat@gmail.com

**Keywords:** ancient DNA, *Bos indicus*, *Bos taurus*, extinction, Southeast Asia, Thailand

## Abstract

In Southeast Asia (SEA), *Bos indicus* (zebu) refers to the traditional cattle, whereas *Bos taurus* (taurine) refers to exotic breeds imported from foreign countries. Notably, a previous study reported that all Thai cattle dating to 3500–1700 years before present (YBP) belonged to the taurine lineage. This suggests that zebu may have been introduced into Thailand at a later date, subsequently replacing the taurine population. In the present study, we analysed 26 cattle remains from nine archaeological sites across Thailand dated to between 3400 and 600 YBP. Taxonomic classification of the specimens was determined using partial D-loop sequences. DNA from four cattle remains obtained from two archaeological sites (Khao Khuram, dated to 1700–1500 YBP, and Sukhothai Historical Park, dated to 850–650 YBP) was successfully amplified and sequenced. Both phylogenetic and haplotype network analyses showed that these remains were grouped in the same haplotype as modern zebu cattle from India, China, and Cambodia. The results suggest that ancient Thai cattle belonged to the zebu lineage and that zebu cattle were introduced into Thailand from India at least 1700 years ago. Furthermore, genetic relationships suggest two potential routes of introduction into Thailand and other SEA countries via overland and maritime routes.

## Simple Summary

*Bos taurus* (taurine) and *Bos indicus* (zebu) are domestic cattle species that originated in the Near East (Middle East) and the Indus Valley (Pakistan, northwest India, and Afghanistan), respectively. Zebu is the predominant traditional cattle lineage in Southeast Asia, whereas taurine cattle are exotic breeds imported from other countries. A previous study suggested that all Thai cattle dating to 3500–1700 years before present (YBP) belonged to the taurine lineage. This suggests that zebu cattle were introduced into Thailand at a later date and subsequently replaced the taurine population. This study analysed 26 cattle specimens collected from nine archaeological sites across Thailand, dating between 3400 and 600 YBP. Species identification was performed using partial mitochondrial DNA sequences obtained from four cattle remains from two archaeological sites, Khao Khuram (dated 1700–1500 YBP) and Sukhothai Historical Park (dated 850–650 YBP). DNA analyses showed that these ancient cattle were grouped with modern zebu cattle from India, China, and Cambodia, suggesting that zebu cattle linked to South Asian lineages had reached Thailand by at least 1700 YBP. The findings provide new insights into domestic animal mobility and transregional exchange networks that link South and Southeast Asia via overland and maritime routes.

## 1. Introduction

Domestic cattle are raised in more than 190 countries globally [1]. They serve vital roles as food sources and in the leather industry, agriculture, transportation, and ritual ceremonies [2,3]. There are two primary domestic cattle species, *Bos taurus* (taurine) and *Bos indicus* (zebu), that are distributed across Europe, the Americas, Australia/Oceania, Africa, and Asia [4]. *B. taurus* originated in the Near East approximately 10,000 YBP and was derived from *Bos primigenius primigenius* (wild aurochs) [5]. *B. indicus* originated in the Indus Valley approximately 8000 YBP and are descended from *B. primigenius namadicus* [6]. Both domestic lineages subsequently expanded globally. At present, most cattle in Europe and East Asia are *B. taurus*, whereas *B. indicus* predominates in South America, Africa, and Southeast Asia (SEA) [7].

*B. taurus* reached Europe and Africa between 7400 and 7000 YBP, followed by the introduction of *B. indicus* into these regions around 3500–2500 YBP [8,9]. In the Americas, *B. taurus* was first introduced into the Caribbean islands from Europe in 1492, with subsequent importations to the mainland occurring multiple times between the 16th and 18th centuries [10,11]. For *B. indicus*, documentation indicates that it was introduced to South America from the Indian subcontinent less than 150 years ago [12]. In East Asia, the earliest evidence of *B. taurus* dates to approximately 5500–5300 YBP in Northeast China [13]. *B. indicus* is currently widely distributed in Southern China. Although the mitochondrial DNA (mtDNA), Y chromosome and autosome from modern cattle suggest that *B. indicus* was imported into China between 5000–3000 YBP [14], no ancient DNA (aDNA) evidence has yet confirmed the exact timeline of its introduction into this region. In SEA, archaeological records indicate that cattle were reared millennia ago. However, only one aDNA study has confirmed that *B. taurus* was reared in Central and Northeastern Thailand between 3500 and 1700 YBP [15]. Given that *B. indicus* is the predominant traditional cattle species in modern SEA, including Thailand, and that *B. taurus* exists primarily as an exotic breed, two critical questions arise: when was *B. indicus* introduced to the region, and how did the ancient *B. taurus* populations become locally extinct?

In this study, we address the first of these questions. This research aimed to identify cattle species from skeletal remains recovered from nine archaeological sites across Thailand, dating between 3400 and 600 YBP. Among these, archaeological evidence associated with Indian trade networks has been discovered at three sites dating between 1700 and 1000 YBP. A partial mitochondrial D-loop region was selected for amplification. The resulting ancient DNA sequences were evaluated using phylogenetic, nucleotide polymorphism, and haplotype network analyses to elucidate the genetic relationships between ancient Thai cattle and *B. taurus* and *B. indicus* populations from other Asian regions. The data obtained from this study provide insights into the history of cattle husbandry and introduction pathways in SEA, particularly within Thailand.

## 2. Materials and Methods

### 2.1. Archaeological Sites and Sample Collection

**Figure 1:**
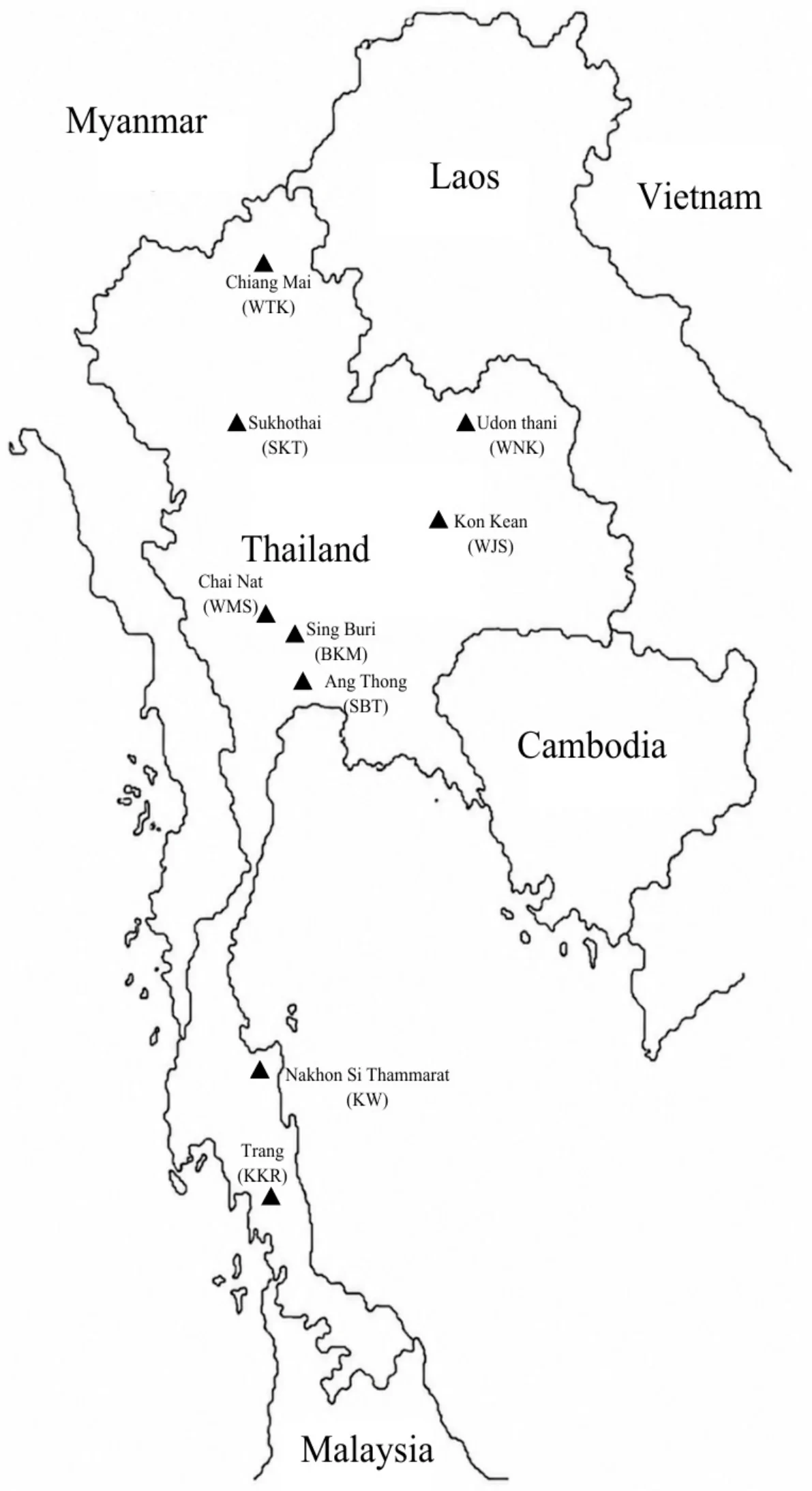
The locations of nine archaeological sites; Wat Nakathewi (WNK), Si Bua Thong (SBT), Wat Jomsri (WJS), Khao Khuram (KKR), Ban Khumuang (BKM), Wiang Thakan (WTK), Kok Wat (KW), Wat Mahathat Sanburi (WMS), and Sukhothai Historical Park (SKT).

A total of 26 cattle remains were collected from nine archaeological sites across Thailand that have been dated to approximately 3400–600 YBP. These sites were Wat Nakathewi (WNK), Si Bua Thong (SBT), Wat Jomsri (WJS), Khao Khuram (KKR), Ban Khumuang (BKM), Wiang Thakan (WTK), Kok Wat (KW), Wat Mahathat Sanburi (WMS) archaeological sites, and Sukhothai Historical Park (SKT) (Figure 1 and Table 1).

The WNK, SBT, and WJS sites, dated to approximately 3400–3200, 3000–2500, and 2400–1800 YBP, respectively, belong to the prehistoric period [16]. KKR represents a transitional period between the Iron Age and the early historical period, dating to approximately 1700–1500 YBP [16,17]. The remaining sites, BKM (1400–1000 YBP), WTK (1300–1000 YBP), KW (1300–1000 YBP), SKT (850–650 YBP), and WMS (700–600 YBP), are assigned to the historic period [16]. Among these nine sites, five (KKR, BKM, KW, SKT, and WMS) have yielded archaeological artifacts linked to India, such as carnelian beads and metal coins featuring Sanskrit inscriptions [16,17,18,19,20,21,22].

### 2.2. DNA Extraction, D-Loop Amplification, and DNA Sequencing

The surfaces of the cattle remains were cleaned using a hand drill before being exposed to ultraviolet (UV) light for 30 min inside a laminar flow hood. The specimens were then ground into powder using a sterilized mortar and pestle. Total DNA was extracted following the protocol in Damgaard et al. (2015) [23]. An extraction blank containing no bone powder was processed simultaneously with each sample batch. The extracted DNA was utilized as a template to amplify the mitochondrial D-loop region using two primer pairs. The first pair consisted of the forward primer (16022–16041): 5′-GCCCCATGCATATAAGCAAG-3′ [24] and the reverse primer (16315–16293): 5′-GGAAAGAATGGACCGTTTTAGAT-3′ [3]. The second pair consisted of the same forward primer and the reverse primer (16178–16159): 5′-CACGCGCATGGTAATTAAG-3′ [24]. PCR amplifications were performed in 50 μL reaction volumes containing 5 μL of 10× PCR buffer, 1.5 μL of 50 mM MgCl_2_, 1 μL of 10 mM dNTPs, 0.5 μL of rabbit serum albumin (Sigma-Aldrich, St. Louis, MO, USA), 0.5 μL of each primer, 0.2 μL of Platinum™ Taq DNA Polymerase (Invitrogen, Carlsbad, CA, USA), 4 μL of DNA template, and DNase-RNase-free water. The PCR cycling conditions were as described in Siripan et al. (2019) [15]. Amplification products were visualized via 2% agarose gel electrophoresis and stained with Prime Juice (Bio-Helix, New Taipei City, Taiwan). Successfully amplified PCR products were purified and subjected to Sanger sequencing using an ABI 3730XL DNA Analyzer (Applied Biosystems, Foster City, CA, USA).

### 2.3. Authentication of Ancient DNA

To prevent exogenous DNA contamination, all procedures, from sample preparation through DNA extraction and PCR setup, were performed in a dedicated laminar flow hood pre-exposed to UV light for 30 min. All equipment was cleaned with 3% H_2_O_2_, then sterilized with double-distilled water, prior to and immediately after use. Sterilized filter pipette tips were used exclusively throughout the extraction and amplification phases to prevent cross-contamination between samples. Negative controls (extraction blanks and PCR controls) were included in each experimental step. The investigators wore protective clothing, face masks, bouffant caps, and disposable gloves at all times during laboratory procedures. Ancient specimens and DNA were kept in the ancient laboratory, whereas modern cattle specimens and PCR products were not allowed in the laboratory. PCR was conducted in a separate laboratory.

### 2.4. Sequence Analyses

Raw DNA sequences were inspected and edited using BioEdit v7.2.5 [25]. Sequences obtained from the same specimen were aligned, and the primer sequences were trimmed. The resulting 116 bp D-loop sequences from ancient Thai cattle were compared against *B. taurus* and *B. indicus* reference sequences from various Asian countries available in the GenBank database. A total of 124 D-loop sequences from both species (Table S1) were included in subsequent analyses.

Phylogenetic trees and haplotype networks were constructed to identify species and evaluate genetic relationships between ancient Thai cattle from this study and reference sequences from Thailand and other countries. *Bos javanicus* was used as the outgroup. A neighbour-joining (NJ) phylogenetic tree was generated in MEGA11 [26] using the Maximum Composite Likelihood (MCL) model [27], while a maximum-likelihood tree was constructed using IQ-TREE v1.6.12 [28] with the HKY+F+G4 model [29]. A median-joining haplotype network was constructed using Hapsolutely 0.2.3 [30] and DnaSP6 [31].

## 3. Results and Discussion

### 3.1. Success of D-Loop Amplification and Sequencing

Twenty-six cattle specimens were used for the amplification of a partial D-loop region. However, only four ancient specimens (KKR1 (PZ513874), KKR2 (PZ513875), KKR3 (PZ513876), and SKT1 (PZ513877)) excavated from two archaeological sites, Khao Khuram and Sukhothai Historical Park, were successfully amplified and sequenced. The overall success rate was 16% (4 out of 26 specimens) (Table 1). However, at the Khao Khuram and Sukhothai sites, the success rates were 100% and 50%, respectively, indicating that DNA preservation at these two sites was better than at the others. Ancient DNA is usually degraded into small fragments of 40–500 bp [32]. Moreover, extreme DNA damage is often observed in tropical regions due to high temperatures and high humidity. Previously, approximately 70% of cattle remains collected from the Ban Chiang archaeological site in northeast Thailand, dating to 3550–2850 YBP, failed to amplify a 157 bp D-loop region [15]. This finding indicated that most ancient DNA in tropical areas, including Thailand, is heavily fragmented into segments of fewer than 160 bp.

Several factors, such as past flooding events, can affect DNA quantity and quality. KKR is located approximately 36 m above sea level, and no flooding events have been reported. Similarly, SKT was surrounded by ancient moats and earthen constructions that protected the ancient city from flooding. However, the other sites, including WJS, BKM, WMS, and KW, had been flooded several times, potentially resulting in severe DNA degradation. Water can destroy DNA through hydrolysis, a major factor causing DNA lesions in ancient specimens [33].

Previously, the most successful DNA extraction and amplification of ancient cattle remains in central and northeast Thailand came from specimens dated 2850–1750 YBP (66.67%; 12 out of 18 specimens), while the oldest specimens dated 3550–2850 YBP showed a lower success rate (28.57%; 8 out of 28 specimens) [15]. Notably, most of the specimens with high success rates were from the Ban Dung site, located in a salt production area [34]. High salinity can create an anhydrous environment in which DNA is well preserved due to limited water access in ancient specimens [35]. Recently, PCR amplification of a 159 bp D-loop region from ancient rhino specimens from two archaeological sites in northeast and central Thailand, dated 3550–2850 and 1250–450 YBP, failed [36]. Similarly, ancient specimens from WNK, the oldest site in this study, dated to between 3400 and 3200 YBP, also failed to yield PCR products for the 159 bp D-loop region. Hence, the age and conditions of archaeological sites may affect the quality and quantity of DNA preserved in ancient remains.

The condition of the specimens is also a significant factor for DNA preservation. Recently, two rhino teeth from Khao Samroi Yod National Park (both dated approximately 100 YBP) were successfully amplified [37]. One extracted from a mandible showed no cracks, and a 301 bp D-loop region was successfully amplified using a single set of primers, while the other, with a small crack on one root, required three sets of primers for nested and semi-nested PCR to produce overlapping products (sizes ranging between 159 and 170 bp) covering the 301 bp D-loop region [37]. In this study, no PCR products could be generated from cattle remains at seven archaeological sites because most of the remains were fragmented. Water and microbes can penetrate specimens through small cracks and destroy the internal DNA. Taken together, these findings indicate that the completeness and age of ancient remains, as well as the conditions at the archaeological sites, affected the success rate of DNA amplification and sequencing in this study.

### 3.2. Phylogenetic Analysis

**Figure 2:**
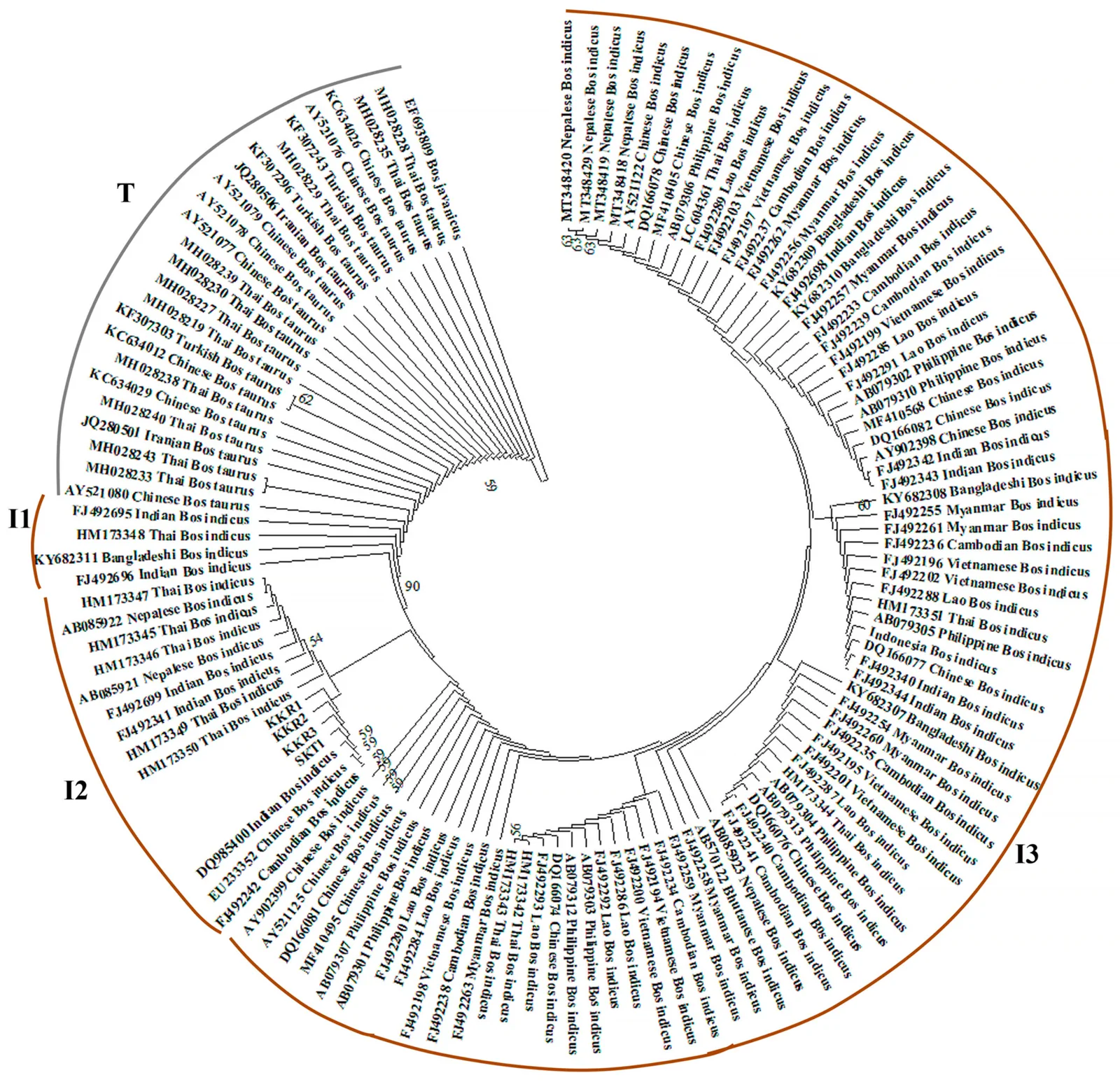
The phylogenetic relationships between the ancient Thai cattle (KKR1, KKR2, KKR3, and SKT1) in this study and available 124 D-loop sequences of *Bos taurus* and *Bos indicus* from the GenBank database. I1, I2, and I3 represent clades of *B. indicus*, while T represents a clade of *B. taurus.* Bootstrap values (only those ≥ 50% are shown), indicating the percentages of 1000 replicates, are presented at each node. *B. javanicus* was used as the outgroup.

The NJ tree (Figure 2) and the ML tree (Figure S1) resolved two distinct groups corresponding to *Bos taurus* (T) and *Bos indicus* (I). The *B. indicus* group was further divided into three subclades, designated I1 to I3. I1 consisted of modern *B. indicus* from Thailand, India, and Bangladesh. The close relationship of the cattle from these countries suggests the introduction of cattle from India to Thailand via a coastal route. I2 comprised two subclades: the ancient Thai samples from this study (KKR1, KKR2, KKR3 and SKT1) and modern *B. indicus* from India, China, and Cambodia; and a subclade comprising modern *B. indicus* from Thailand, India, and Nepal, supported by bootstrap values of 59% and 54%, respectively. The clustering of KKR1, KKR2, KKR3, and SKT1 with *B. indicus* indicates that all ancient Thai specimens belonged to *B. indicus*, demonstrating a close genetic relationship. Moreover, grouping these ancient specimens with five modern Thai *B. indicus* individuals (HM173345–HM173347, HM173349–HM173350) suggests a close maternal affinity. The ancient Thai cattle sequences were highly similar to those of modern *B. indicus* from India (DQ985400), China (EU233352), and Cambodia (FJ492242). The results suggest that ancient Thai *B. indicus* shared a common ancestry with these modern populations. Furthermore, the close relationship among lineages from India, China, and Mainland Southeast Asia (MSEA) suggests that *B. indicus* may have been transported from India to Thailand via China using an overland Silk Road. Lastly, I3 consisted of *B. indicus* from Thailand, other MSEA countries, Island Southeast Asia (ISEA; Indonesia and the Philippines), China, and South Asia (Bangladesh, India, and Nepal). This clade demonstrated a close relationship among *B. indicus* from MSEA, ISEA, South Asia, and China. Given that MSEA, including Thailand, serves as a maritime trade station connecting China and India, these findings suggest a potential maritime trade route for the introduction of cattle from India to Thailand.

During the Early Historic period, the Thai-Malay Peninsula became an important interaction zone linking India and Southeast Asia through maritime and trans-peninsular trade networks. Archaeological evidence from sites such as Khao Sam Kaeo, Phu Khao Thong, and Khlong Thom indicates intensive exchange involving imported beads, coins, religious objects, and other prestige goods associated with India and Roman trade [16,38,39]. In this context, the close genetic relationship between Thai cattle and those from Island Southeast Asia (ISEA) suggests that the dispersal of *B. indicus* may have been connected to these maritime exchange systems. Archaeological evidence indicates regular interactions between India and Southeast Asia in antiquity. Maritime trade routes extending from China through Southeast Asia to India and the Mediterranean were established by approximately 2300 YBP [40]. For example, the Pak Klong Kluai shipwreck in Ranong Province has been dated to approximately 2100 YBP [41]. The vessel was constructed using mortise-and-tenon techniques commonly associated with Indian Ocean shipbuilding traditions. Nearby, the Phu Khao Thong archaeological site has yielded intaglios depicting Indian-style humped bull figures, as well as imported beads, Roman coins, and Brahmi inscriptions dating between the 1st century BCE and 3rd century CE [39]. Together, these finds reflect sustained maritime interaction between South Asia and Southeast Asia over 2000 years ago. Similar findings have also been reported in neighbouring Southeast Asian countries. At Phum Snay and Phum Sophy in Cambodia, bovine remains recovered from burial contexts together with trade goods such as carnelian beads point to connections between South and SEA during the Iron Age and the Early Historic period [42,43]. In Vietnam and Peninsular Malaysia, there is limited archaeozoological evidence for cattle husbandry. However, Early Historic materials from these regions include Indian-style humped cattle imagery associated with Hindu traditions, particularly within the Óc Eo cultural sphere [44,45]. Taken together, these archaeological data further support the connection between MSEA and ISEA through maritime trade and suggest that *B. indicus* may have spread into both regions through maritime and overland exchange networks.

In the present study, carnelian beads—among the earliest and most widespread maritime trade commodities from India—have been excavated at the Khao Khuram site, confirming historical contact between India and ancient Thai communities via maritime trade. Taken together, our results suggest that *B. indicus* was introduced into Thailand through two primary pathways: an overland Silk Road route via Bangladesh and Myanmar, and a maritime trade route. Furthermore, *B. indicus* from Thailand exhibited a close genetic relationship with Chinese populations in all five clades. Consistent with previous findings, multiple ancient DNA studies have documented the introduction of pigs, domestic cattle (*B. taurus*), and *japonica* rice from China to Thailand during the ancient period between 3500 and 1500 YBP [15,46,47,48]. Additional archaeological evidence suggests that *B. indicus* from India may have been introduced into South China and Southeast Asia between 3500 and 2500 YBP [49].

### 3.3. Nucleotide Polymorphism

The analysed cattle sequences were classified into 29 distinct haplotypes, with H1–H16 and H17–H29 belonging to *B. indicus* and *B. taurus*, respectively (Table S2). A total of 34 polymorphic sites were detected, comprising 32 transitions, one transversion, and one insertion/deletion (indel). *B. taurus* was differentiated from *B. indicus* by 11 nucleotide positions at sites 17, 41, 61, 68, 75, 76, 80, 81, 96, 97, and 102. Among these, 10 were transition substitutions: T to C (at position 17), A to G (at positions 41, 61, and 76), and C to T (at positions 68, 75, 81, 96, and 97). The remaining nucleotide position at site 102 was characterized by a deletion (gap) in *B. indicus* and an adenine (A) in *B. taurus*. Among the *B. indicus* populations, Thailand and India exhibited the highest haplotype diversity (seven haplotypes each), followed by Nepal (six haplotypes) and Cambodia (three haplotypes).

All ancient cattle specimens from this study clustered into a single haplotype, H1, together with *B. indicus* from India, China, and Cambodia. This result confirmed that the ancient remains belonged to *B. indicus*. Although the ancient Thai *B. indicus* specimens did not share a haplotype with modern Thai *B. indicus*, they displayed only a few nucleotide differences, supporting a very close genetic relationship. Modern Thai cattle were distributed across seven haplotypes. Two of these (H2 and H3) were shared with modern *B. indicus* from other countries, while the remaining five formed unique, local haplotypes. Consistent with a previous genome-wide study, a distinct Southeast Asian ancestry has been identified in indigenous Thai *B. indicus*, particularly in cattle populations from the southern region [50]. These findings imply that a distinct genetic pool of Thai *B. indicus* has evolved in isolation since its introduction to Thailand.

### 3.4. Haplotype Network Analyses

**Figure 3:**
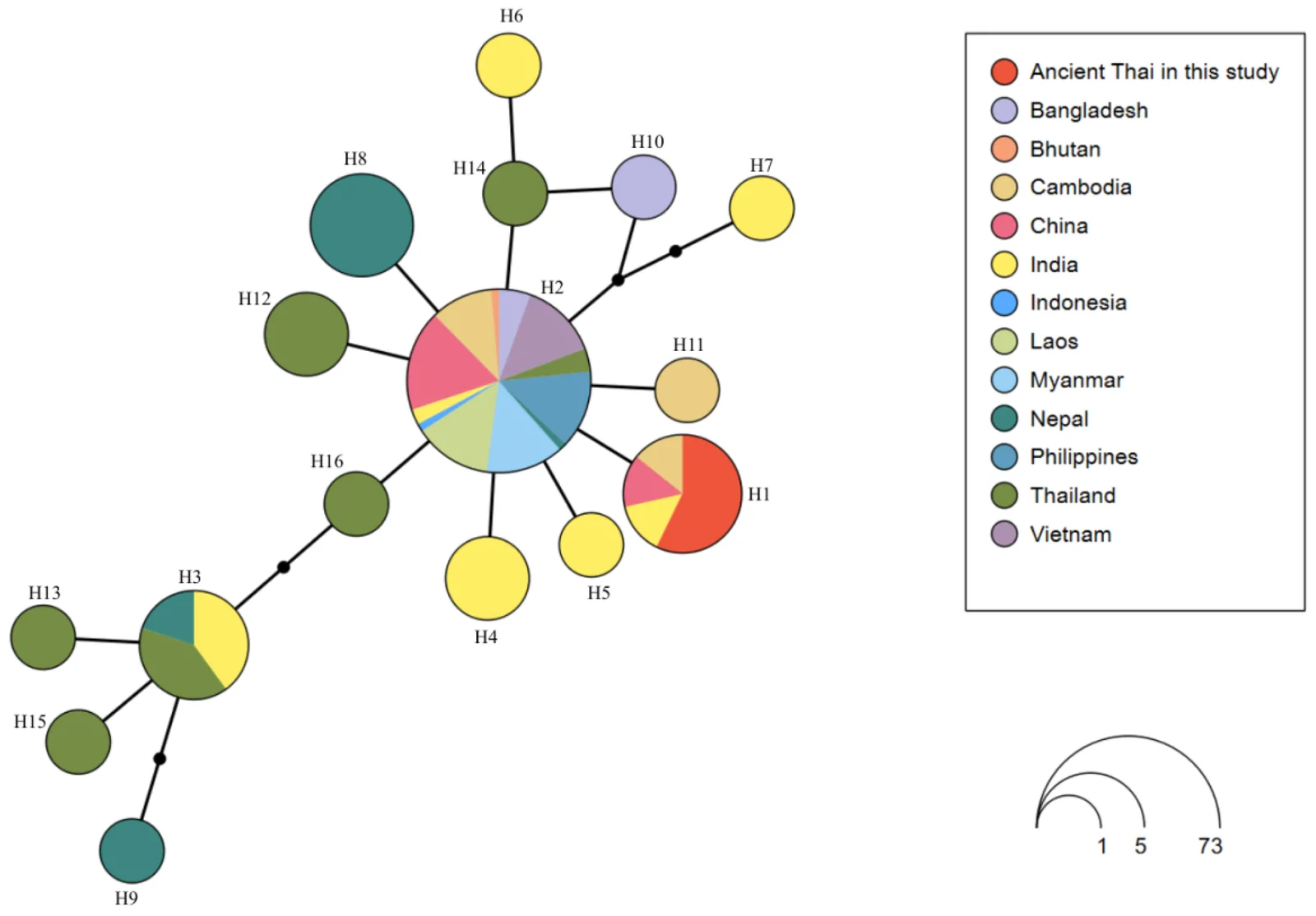
A haplotype network of D-loop sequences from ancient Thai cattle in this study (H1) and modern *B. indicus* from South Asia, East Asia, MSEA, and ISEA. The size of each circle is proportional to the number of sequences. Different cattle populations are shown by separate colours. The black dots that cross the lines on each branch indicate the number of mutations.

The median-joining network analysis was performed using only *B. indicus* sequences (Figure 3). The network exhibited a star-like topology centred on H2 as the predominant ancestral haplotype. This core haplotype contained 73 individuals from 12 countries spanning South Asia (India, Bangladesh, Nepal, and Bhutan), East Asia (China), Mainland SEA (Myanmar, Cambodia, Vietnam, Laos, and Thailand), and Island SEA (Indonesia and the Philippines). The remaining haplotypes diverged from H2 by only one to four nucleotide substitutions. All ancient Thai cattle from this study (KKR1, KKR2, KKR3, and SKT1) belonged to haplotype H1. Furthermore, the inclusion of modern *B. indicus* from India, China, and Cambodia within this haplotype indicates that *B. indicus* from Thailand, India, China, and Cambodia share a common maternal ancestor, and that this lineage has been continuously maintained in SEA from 1700 YBP to the present day.

Interestingly, *B. indicus* from Thailand was distributed across seven haplotypes (H2–H3, H12–H16), with H3, H13, H15, and H16 forming distinct lineages diverging from the central H2 haplotype. Haplotype H3 comprised *B. indicus* from Thailand, China, and Nepal. This genetic affinity supports an introduction pathway of *B. indicus* from India into Thailand and other MSEA nations via Nepal and China. Conversely, the co-clustering of Island SEA and Mainland SEA populations points toward a secondary wave of cattle importation through maritime trade networks.

The results from this study suggest that partial D-loop sequences can be used to identify cattle species. Moreover, the phylogenetic and haplotype network analyses have revealed genetic relationships between ancient Thai cattle and those from other countries, suggesting potential routes of introduction of *B. indicus* in ancient times. However, bootstrap values and the number of polymorphic sites detected among *B. indicus* are quite low for resolving population-level relationships. Hence, longer sequences, the complete D-loop, or whole mitochondrial DNA should be used for further study if possible. In addition, the nuclear genome should be included in ancient cattle studies to determine whether hybridisation occurred between *B. indicus* and *B. taurus* and SEA in ancient times.

Although domestic cattle have been raised in SEA for millennia, no previous ancient DNA studies have definitively confirmed the exact arrival timeline of *B. indicus* in the region, including Thailand. Rich archaeological assemblages linked to Indian trade have been uncovered at the Khao Sam Kaeo (KSK) site in Southern Thailand, dating to 2400–1500 YBP [38]. Furthermore, silver coins depicting a humped bull and Sanskrit inscriptions have been excavated in Dvaravati cities across Central Thailand, dating to approximately 1300 YBP [51,52]. The Dvaravati culture, heavily influenced by Indian religion, art, and language, flourished throughout Central and Northeast Thailand between 1500 and 900 YBP [53]. Previous studies have suggested that religious expansion from India reached the eastern regions of the country over 2000 YBP [54], potentially driving the dispersal of *B. indicus* into SEA [2]. Moreover, archaeological remnants of an early Buddhist shrine and miniature stupas dating to approximately 1500–1400 YBP have been documented near the Khao Khuram site, confirming the spread of Indian religious practices during the early historic period [16,17]. Additionally, a burial containing glass beads alongside a complete cattle skeleton at the KW archaeological site in Nakhon Si Thammarat Province suggests the presence of Brahmanical rituals in Southern Thailand between 1300 and 1000 YBP [22]. Two earthenware vessels containing Arabic silver coins were also discovered at KW, indicating robust maritime commerce between Thailand and the Middle East [22]. Collectively, these diverse archaeological records confirm intensive interaction between India and Thailand, particularly in the southern peninsula, as early as 2400 YBP. Furthermore, a modern DNA study of *B. indicus* using the mitogenome, Y chromosomes, and autosomes found that the cattle species was introduced into East Asia between 5000 and 3000 YBP via SEA along a coastal route [14]. Hence, it is possible that *B. indicus* was introduced into Thailand and the SEA between 5000 and 2400 YBP. Our ancient DNA evidence demonstrates that *B. indicus* had arrived in this region by at least 1700 YBP. The absence of successful amplification from older sites (WNK, SBT, and WJS) dated between 3400 and 1800 YBP does not rule out the absence of *B. indicus* at those times; it may only reflect poor DNA preservation. To determine whether cattle were introduced into Thailand prior to 1700 YBP, ancient specimens from archaeological sites older than 1700 YBP should be included in further studies. Remarkably, the presence of unique haplotypes in extant Thai *B. indicus* indicates that a distinct, localized gene pool has evolved over a long period, culminating in a unique Southeast Asian lineage. Further studies of pathogens in ancient cattle remains excavated from archaeological sites across broad age ranges throughout the country could provide insight into how ancient *B. taurus* populations became extinct.

## 4. Conclusions

In this study, the success rate of ancient DNA amplification and sequencing was 16% from four cattle specimens from two archaeological sites. The results indicated extreme DNA damage that is often observed in tropical regions. Notably, this study provides the first ancient DNA evidence demonstrating that *B. indicus* was raised in Thailand as early as 1700 YBP. The simultaneous confirmation of both *B. indicus* and *B. taurus* in the country around 1700 YBP suggests that these two cattle lineages co-existed in the country during historical times. Phylogenetic and haplotype network analyses, as well as archaeological evidence, support possible pathways for the introduction of *B. indicus* from India into Thailand, particularly via the maritime trade route. Because only four specimens from two archaeological sites were successfully amplified and sequenced in this study, further ancient DNA research with a larger sample size from broader archaeological contexts across Thailand and neighbouring SEA nations is required to fully elucidate the husbandry history and complex migratory routes of *B. indicus* in this region. The introduction of zebu cattle into Thailand should not be viewed solely as a biological event, but rather as part of broader historical transformations associated with the rise in maritime trade, early state formation, and long-distance cultural interactions across the Indian Ocean and Mainland Southeast Asia.

## Figures and Tables

**Table 1:** Details of ancient specimens and results of D-loop amplification and sequencing.

Lab Code	Skeletal Element	Archaeological Site, Province	Age (YBP)	Dating Method *	Period	Successful Amplifications and Sequencing
WNK1	Long bone	Wat Nakathewi, Udon Thani	3400–3200	AMS of the artifact found nearby	Prehistoric	No
WNK2	Metacarpal					No
WNK3	Humerus					No
WNK4	Femur					No
WNK5	Mandible					No
SBT1 SBT2	Grand cuneiform Long bone	Si Bua Thong, Ang Thong	3000–2500	Relative dating based on pottery pattern found nearby	Prehistoric	No No
WJS1	Radius	Wat Jomsri, Khon Kean	2400–1800	AMS of the artifact found nearby	Prehistoric	No
WJS2	Long bone					No
WJS3	Metacarpal					No
WJS4	Radius					No
KKR1	Femur	Khao Khuram, Trang	1700–1500	AMS of the artifact found nearby	Late prehistoric- early historic	Yes
KKR2	Phalange					Yes
KKR3	Tooth					Yes
BKM1	Tibia	Ban Khumuang, Sing Buri	1400–1000	Relative dating based on Dvaravati-period artifacts	Historic (Dvaravati)	No
BKM2	Femur					No
BKM3	Radius					No
WTK1	Radius	Wiang Thakan, Chiang Mai	1300–1000	AMS of the artifact found nearby	Historic	No
WTK2	Scapula					No
WTK3	Radius					No
KW1	Femur	Kok Wat, Nakhon Si Thammarat	1300–1000	TL of the artifact found nearby	Historic	No
SKT1	Metacarpal	Sukhothai Historical Park, Sukhothai	850–650	Relative dating based on Sukhothai ceramics found nearby	Historic	Yes
SKT2	Metacarpal					No
WMS1	Long bone	Wat Mahatat Sanburi, Chai Nat	700–600	AMS of the artifact found nearby	Historic	No
WMS2	Radius					No
WMS3	Metatarsus					No

* AMS = Accelerator Mass Spectrometry; TL = Thermoluminescence.

## Data Availability

The original contributions presented in this study are included in the article/Supplementary Materials. Further inquiries can be directed to the corresponding author.

## Supplementary Materials

The following supporting information can be downloaded at https://www.mdpi.com/article/10.3390/ani16121904/s1, Table S1: D-loop sequences of *Bos* species retrieved from GenBank database and used for nucleotide polymorphism, Neighbor-Joining and haplotype network analyses in this study; Table S2: Nucleotide polymorphism in a D-loop region in modern and ancient cattle; Figure S1: The maximum-likelihood relationship between the ancient Thai cattle (KKR1, KKR2, KKR3 and SKT1) in this study and available 124 D-loop sequences of *Bos taurus* and *Bos indicus* from GenBank database. Refs. [55,56,57,58,59,60,61,62,63,64,65,66,67,68] are cited in Supplementary Materials.
