# Supplementary material for: The First Ancient DNA Evidence of Zebu Husbandry in Thailand During the Prehistoric Through the Historic Periods
Source: Animals (Basel). 2026 Jun 19;16(12):1904. doi: 10.3390/ani16121904 (PMC13295790; doi:10.3390/ani16121904)
Supplement: Supplementary file 1 [file animals-16-01904-s001.zip › animals-4362823-supplementary.pdf]

## Supplementary

**Table S1** D-loop sequences of *Bos* species retrieved from GenBank database and used for nucleotide polymorphism, Neighbor-Joining and haplotype network analyses in this study

| Species | Country | Accession No. | Reference |
|---|---|---|---|
| *B. indicus* | India | FJ492340–FJ492344, FJ492695–FJ492696, FJ492698–FJ492699 | Chen et al., 2010 |
| | | DQ985400 | Bhuiyan et al., 2007 |
| | Nepal | MT348418–MT348420, MT348429 | Gorkhali et al., 2020 |
| | | AB085921–AB085923 | Fujise et al., 2003 |
| | Bangladesh | KY682307–KY682311 | Gao et al., 2017 |
| | Bhutan | AB570122 | Dorji et al., 2010 |
| | Myanmar | FJ492254–FJ492263 | Chen et al., 2010 |
| | Cambodia | FJ492233–FJ492242 | Chen et al., 2010 |
| | Vietnam | FJ492194–FJ492203 | Chen et al., 2010 |
| | Laos | FJ492284–FJ492293 | Chen et al., 2010 |
| | Thailand (modern) | HM173342–HM173351 | Siripan et al., 2019 |
| | | LC604361 | Ariyaraphong et al., 2021 |
| | Philippines | AB079301–AB079307, AB079310, AB079312–AB079313 | Komatsu et al., 2004 |
| | Indonesia | - | Putri et al., 2019 |
| | China | EU233352 | Gao et al., 2017 |
| | | MF410405, MF410495, MF410568 | Li et al. 2018 |
| | | DQ166074, DQ166076–DQ166078, DQ166081–DQ166082 | Lei et al., 2006 |
| | | AY521122, AY521125, AY902398–AY902399 | Lei et al., 2006 |
| *B. taurus* | Thailand (ancient) | MH028219, MH028227–MH028230, MH028233, MH028235, MH028238–MH028240, MH028243 | Siripan et al., 2019 |
| | China | AY521076–AY521080 | Lei et al., 2006 |
| | | KC634012, KC634026, KC634029 | Cai et al., 2014 |
| | Iran | JQ280501, JQ280506 | Bollongino et al., 2012 |
| | Turkey | KF307243, KF307296, KF307303 | Scheu et al., 2013 |
| *B. javanicus* | | EF693809 | Hassanin and Ropiquet, 2007 |

## References


Ariyaraphong, N.; Laopichienpong, N.; Singchat, W.; Panthum, T.; Ahmad, S.F.; Jattawa, D.; Duengkae, P.; Muangmai, N.; Suwanasopee, T.; Koonawootrittriron, S.; et al. High-level gene flow restricts genetic differentiation in dairy cattle populations in Thailand: Insights from large-scale mt D-Loop Sequencing. Animals 2021, 11, 1680.

Bhuiyan, M.; Bhuiyan, A.K.F.H.; Yoon, D.; Jeon, J.; Park, C.S.; Lee, J.H. Mitochondrial DNA diversity and origin of red Chittagong cattle. Asian-Australas. J. Anim. Sci. 2007, 20, 1478–1484.

Bonfiglio, S.; Ginja, C.; De Gaetano, A.; Achilli, A.; Olivieri, A.; Colli, L.; Tesfaye, K.; Agha, S.H.; Gama, L.T.; Cattonaro, F.; et al. Origin and spread of *Bos taurus*: new clues from mitochondrial genomes belonging to haplogroup T1. PLoS ONE 2012, 7, e39011.

Cai, D.; Sun, Y.; Tang, Z.; Hu, S.; Li, W.; Zhao, X.; Xiang, H.; Zhou, H. The origins of Chinese domestic cattle as revealed by ancient DNA analysis. J. Archaeol. Sci. 2014, 41, 423–434.

Chen, S.; Lin, B.Z.; Baig, M.; Mitra, B.; Lopes, R.J.; Santos, A.M.; Magee, D.A.; Azevedo, M.; Tarroso, P.; Sasazaki, S.; et al. Zebu cattle are an exclusive legacy of the South Asia Neolithic. Mol. Biol. Evol. 2010, 27, 1–6.

Dorji, T.; Kaneda, M.; Lin, B.Z.; Takahashi, A.; Oyama, K.; Sasazaki, S.; Yamamoto, Y.; Kawamoto, Y.; Mannen, H. Mitochondrial DNA variation and genetic construction of indigenous cattle population in Bhutan. J. Anim. Genet. 2010, 38, 77–81.

Fujise, H.; Murakami, M.; Devkota, B.; Dhakal, I.; Takeda, K.; Hanada, H.; Fujitani, H.; Sasaki, M.; Kobayashi, K. Breeding distribution and maternal genetic lineages in Lulu, a dwarf cattle population in Nepal. Anim. Sci. J. 2003, 74, 1–5.

Gao, Y.; Gautier, M.; Ding, X.; Zhang, H.; Wang, Y.; Wang, X.; Faruque, M.O.; Li, J.; Ye, S.; Gou, X.; et al. Species composition and environmental adaptation of indigenous Chinese cattle. Sci. Rep. 2017, 7, 16103.

Gorkhali, N.; Dhakal, A.; Sapkota, S.; Sherpa, C.; Pokhrel, B.; Kolachhapati, M.; Bhattarai, N. Mitochondrial DNA polymorphisms in Nepalese Achhami cattle. Bangladesh J. Anim. Sci. 2020, 49, 22–28.

Hassanin, A.; Ropiquet, A. Resolving a zoological mystery: the kouprey is a real species. Proc. R. Soc. B Biol. Sci. 2007, 274, 2849–2855

Komatsu, M.; Yasuda, Y.; Matias, J.M.; Niibayashi, T.; Abe-Nishimura, A.; Kojima, T.; Oshima, K.; Takeda, H.; Hasegawa, K.; Abe, S.; et al. Mitochondrial DNA polymorphisms of D-loop and three coding regions (ND2, ND4, ND5) in three Philippine native cattle: Indicus and taurus maternal lineages. Nihon Chikusan Gakkaiho 2004, 75, 363–378.

Lai, S.J.; Liu, Y.P.; Liu, Y.X.; Li, X.W.; Yao, Y.G. Genetic diversity and origin of Chinese cattle revealed by mtDNA D-loop sequence variation. Mol. Phylogenet. Evol. 2006, 38, 146–154.

Li, R.; Li, C.; Liu, H.; Zeng, B.; Xiao, H.; Chen, S. Mitochondrial diversity and phylogeographic structure of native cattle breeds from Yunnan, Southwestern China. Livest. Sci. 2018, 214, 129–134.

Putri, A.; Farajallah, A.; Perwitasari, D. The origin of pesisir cattle based on D-loop mitochondrial DNA. Biodiversitas J. Biol. Divers. 2019, 20, 2603–2608.

Scheu, A.; Bollongino, R.; Tresset, A.; Vigne, J.D.; Cakirlar, C.; Benecke, N.; Burger, J. The arrival of the first domesticated cattle in Europe - an ancient DNA perspective. In Proceedings of the International

Workgroup for Domestic Animal Bioarchaeology; Johannes Gutenberg-Universität: Mainz, Germany, 2013.

Siripan, S.; Wonnapinij, P.; Auetrakulvit, P.; Wangthongchaicharoen, N.; Surat, W. Origin of prehistoric cattle excavated from four archaeological sites in central and northeastern Thailand. Mitochondrial DNA A DNA Mapp. Seq. Anal. 2019, 30, 609–617.

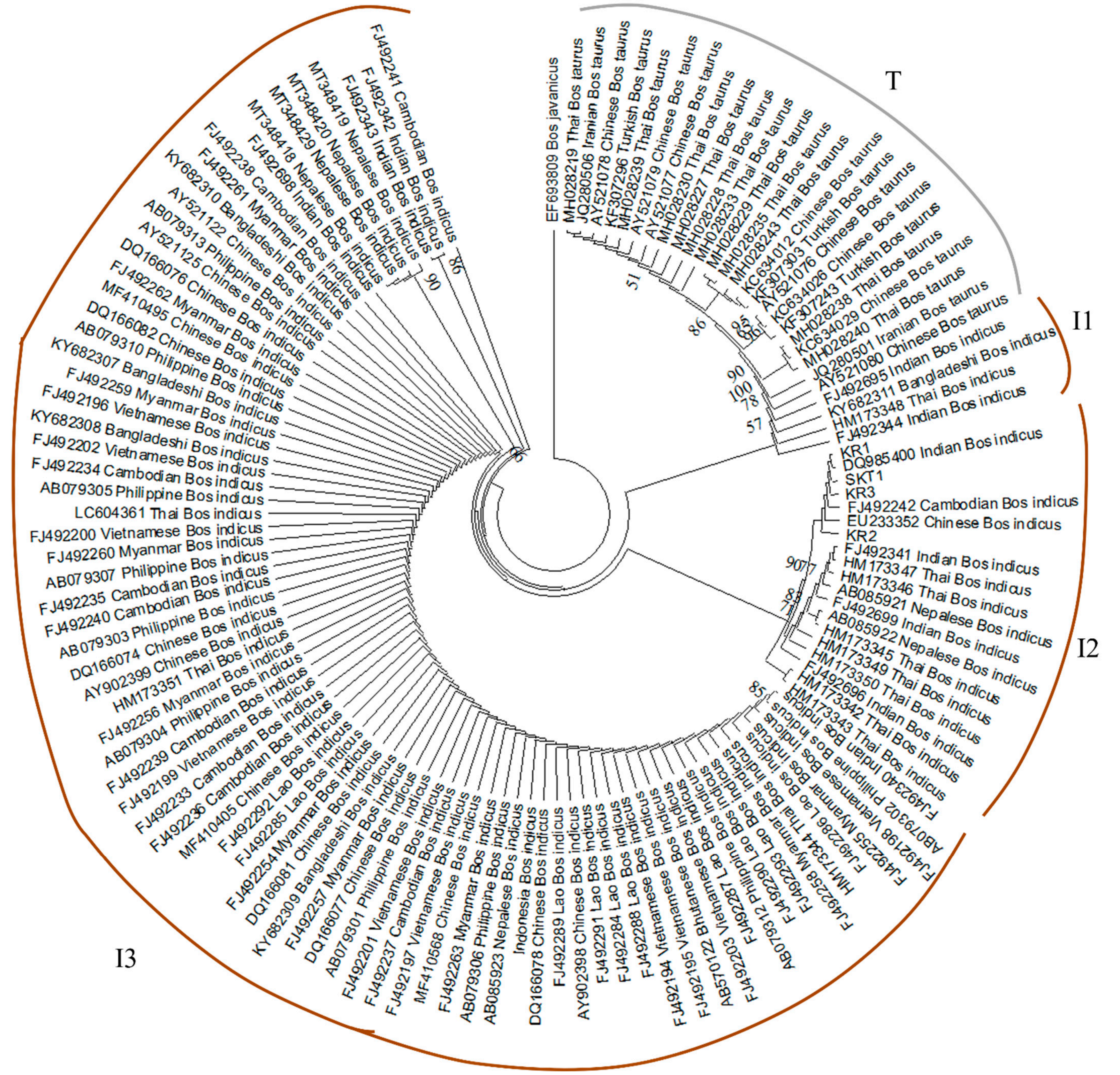


**Figure S1** The maximum-likelihood relationship between the ancient Thai cattle (KKR1, KKR2, KKR3 and SKT1) in this study and available 124 D-loop sequences of *Bos taurus* and *Bos indicus* from GenBank database. I1, I2 and I3 represent three subclades of *B. indicus*, while T represents a clade of *B. taurus*. Bootstrap values (only ≥ 50%) showing in the percentage of 1,000 replicates are presented at each node. *B. javanicus* was used as outgroup.

**<u>Table S2</u>** Nucleotide polymorphism in a D-loop region in modern and ancient cattle

| Haplotype | Nucleotide Position | | | | | | | | | | | | | | | | | | Location * | Species |
|---|---|---|---|---|---|---|---|---|---|---|---|---|---|---|---|---|---|---|---|---|
| | 1 | 8 | 9 | 10 | 11 | 12 | 16 | 17 | 26 | 33 | 41 | 43 | 44 | 52 | 58 | 61 | 67 | 68 | | |
| H1 | T | T | C | T | C | T | A | T | A | C | A | T | T | G | T | A | T | C | Ancient TH in This study, IN, CA, CH | |
| H2 | . | . | . | . | . | . | . | . | . | . | . | . | . | . | . | . | . | . | IN, NE, BA, BU, MY, CA, VI, LA, IND, CH, Modern TH, PH, IND | |
| H3 | . | . | . | . | . | . | . | . | . | . | . | C | C | . | . | . | . | . | IN, NE, Modern TH | |
| H4 | . | . | . | . | . | . | . | . | . | . | . | . | . | . | . | . | . | . | IN | |
| H5 | . | . | . | . | . | . | . | . | . | . | . | . | . | . | C | . | . | . | IN | |
| H6 | . | . | . | . | . | . | . | . | . | T | . | C | . | . | . | . | . | . | IN | |
| H7 | . | . | T | . | . | . | . | . | . | . | . | . | . | . | . | . | . | . | IN | *Bos indicus* |
| H8 | . | . | . | C | . | . | . | . | . | . | . | . | . | . | . | . | . | . | NE | |
| H9 | . | C | . | . | . | . | . | . | G | . | . | C | C | . | . | . | . | . | NE | |
| H10 | . | . | T | . | . | . | . | . | . | T | . | . | . | . | . | . | . | . | BA | |
| H11 | . | . | . | . | . | C | . | . | . | . | . | . | . | . | . | . | . | . | CA | |
| H12 | . | C | . | . | . | . | . | . | . | . | . | . | . | . | . | . | . | . | Modern TH | |
| H13 | . | . | . | . | . | . | . | . | . | . | . | C | C | . | . | . | . | . | Modern TH | |
| H14 | . | . | . | . | . | . | . | . | . | T | . | . | . | . | . | . | . | . | Modern TH | |
| H15 | . | . | . | . | . | . | . | . | . | . | . | C | C | . | . | . | . | . | Modern TH | |
| H16 | . | . | . | . | . | . | . | . | . | . | . | . | . | . | . | . | . | . | Modern TH | |
| H17 | . | C | . | . | . | . | G | C | . | T | G | C | . | . | . | G | . | T | Ancient TH, CH | |
| H18 | . | C | . | . | . | . | G | C | . | T | G | C | . | . | . | G | . | T | Ancient TH | |
| H19 | . | C | . | . | T | . | G | C | . | T | G | C | . | . | . | G | . | T | Ancient TH | |
| H20 | . | C | . | . | . | . | G | C | . | T | G | C | . | . | . | G | . | T | Ancient TH | |
| H21 | . | C | . | . | . | . | G | C | G | T | G | C | . | . | . | G | . | T | Ancient TH | |
| H22 | . | C | . | . | . | . | G | C | . | T | G | C | . | . | . | G | . | T | Ancient TH | *Bos taurus* |
| H23 | . | C | . | . | . | . | G | C | . | T | G | C | . | . | . | G | . | T | Ancient TH, CH | |
| H24 | . | C | . | . | . | . | . | C | . | T | G | C | . | . | . | G | . | T | Ancient TH | |
| H25 | C | C | . | . | . | . | G | C | . | T | G | C | . | A | . | G | . | T | CH | |
| H26 | . | . | . | . | . | . | G | C | . | T | G | C | . | . | . | G | . | T | CH | |
| H27 | . | C | . | . | . | . | C | C | . | T | G | C | . | . | . | G | . | T | CH, TU | |
| H28 | . | C | T | . | . | . | C | C | . | T | G | C | . | . | . | G | . | T | IR | |
| H29 | . | C | . | . | . | . | C | C | . | T | G | C | . | . | . | G | C | T | TU | |

* India (IN), Indonesia (IND), Myanmar (MY), Cambodia (CA), Vietnam (VI), Laos (LA), Thailand (TH), Philippines (PH), China (CH), Bhutan (BU), Nepal (NE), Bangladesh (BA), Turkey (TU), Iran (IR). Dots (.) indicate identical nucleotides to the sequence in the first line.

**Table S2** Nucleotide polymorphism in a D-loop region in modern and ancient cattle (continued)

| Haplotype | Nucleotide Position | | | | | | | | | | | | | | | | | Location * | Species |
|---|---|---|---|---|---|---|---|---|---|---|---|---|---|---|---|---|---|---|---|
| | 72 | 75 | 76 | 77 | 78 | 80 | 81 | 85 | 86 | 88 | 89 | 92 | 96 | 97 | 100 | 102 | 106 | | |
| H1 | T | C | A | A | C | A | C | T | C | A | C | T | C | C | T | - | C | Ancient TH in This study, IN, CA, CH | |
| H2 | C | . | . | . | . | . | . | . | . | . | . | . | . | . | . | - | . | IN, NE, BA, BU, MY, CA, VI, LA, IND, CH, Modern TH, PH, IND | |
| H3 | C | . | . | . | . | . | . | . | . | . | . | . | . | . | C | - | . | IN, NE, Modern TH | |
| H4 | C | . | . | G | . | . | . | . | . | . | . | . | . | . | . | - | . | IN | |
| H5 | C | . | . | . | . | . | . | . | . | . | . | . | . | . | . | - | . | IN | |
| H6 | C | . | . | . | . | . | . | . | . | . | . | . | . | . | . | - | . | IN | |
| H7 | C | . | . | . | . | . | . | C | . | G | . | . | . | . | . | - | . | IN | *Bos indicus* |
| H8 | C | . | . | . | . | . | . | . | . | . | . | . | . | . | . | - | . | NE | |
| H9 | C | . | . | . | . | . | . | . | . | . | . | . | . | . | C | - | . | NE | |
| H10 | C | . | . | . | . | . | . | . | . | . | . | . | . | . | . | - | . | BA | |
| H11 | C | . | . | . | . | . | . | . | . | . | . | . | . | . | . | - | . | CA | |
| H12 | C | . | . | . | . | . | . | . | . | . | . | . | . | . | . | - | . | Modern TH | |
| H13 | C | . | . | . | . | . | . | . | . | . | . | . | . | . | C | - | T | Modern TH | |
| H14 | C | . | . | . | . | . | . | . | . | . | . | . | . | . | . | - | . | Modern TH | |
| H15 | . | . | . | . | . | . | . | . | . | . | . | . | . | . | C | - | . | Modern TH | |
| H16 | C | . | . | . | . | . | . | . | . | . | . | . | . | . | C | - | . | Modern TH | |
| H17 | . | T | G | . | T | G | T | . | . | . | T | . | T | T | . | A | T | Ancient TH, CH | |
| H18 | . | T | G | . | T | G | T | . | A | . | T | . | T | T | . | A | T | Ancient TH | |
| H19 | . | T | G | . | T | G | T | . | . | . | T | . | T | T | . | A | T | Ancient TH | |
| H20 | . | T | G | . | T | G | T | . | . | . | . | . | T | T | . | A | T | Ancient TH | |
| H21 | . | T | G | . | T | G | T | . | . | . | T | . | T | T | C | A | T | Ancient TH | |
| H22 | . | T | G | . | . | G | T | . | T | . | T | . | T | T | . | A | T | Ancient TH | *Bos taurus* |
| H23 | . | T | G | . | . | G | T | . | . | . | T | . | T | T | . | A | T | Ancient TH, CH | |
| H24 | . | T | G | . | T | G | T | . | . | . | T | . | T | T | . | A | T | Ancient TH | |
| H25 | . | T | G | . | T | G | T | . | . | . | T | . | T | T | . | A | T | CH | |
| H26 | . | T | G | . | . | G | T | . | . | . | T | . | T | T | . | A | T | CH | |
| H27 | . | T | G | . | T | G | T | . | . | . | T | . | T | T | . | A | T | CH, TU | |
| H28 | C | T | G | . | . | G | T | . | . | . | T | C | T | T | . | A | T | IR | |
| H29 | . | T | G | . | T | G | T | . | . | . | T | . | T | T | . | A | T | TU | |

* India (IN), Indonesia (IND), Myanmar (MY), Cambodia (CA), Vietnam (VI), Laos (LA), Thailand (TH), Philippines (PH), China (CH), Bhutan (BU), Nepal (NE), Bangladesh (BA), Turkey (TU), Iran (IR). Dots (.) indicate identical nucleotides to the sequence in the first line; Gaps (-) indicate an insertion/deletion.
